# How Bird Necks Get Naked

**DOI:** 10.1371/journal.pbio.1001029

**Published:** 2011-03-15

**Authors:** Kira Heller

**Affiliations:** Freelance Science Writer, Oakland, California, United States of America

**Figure:**
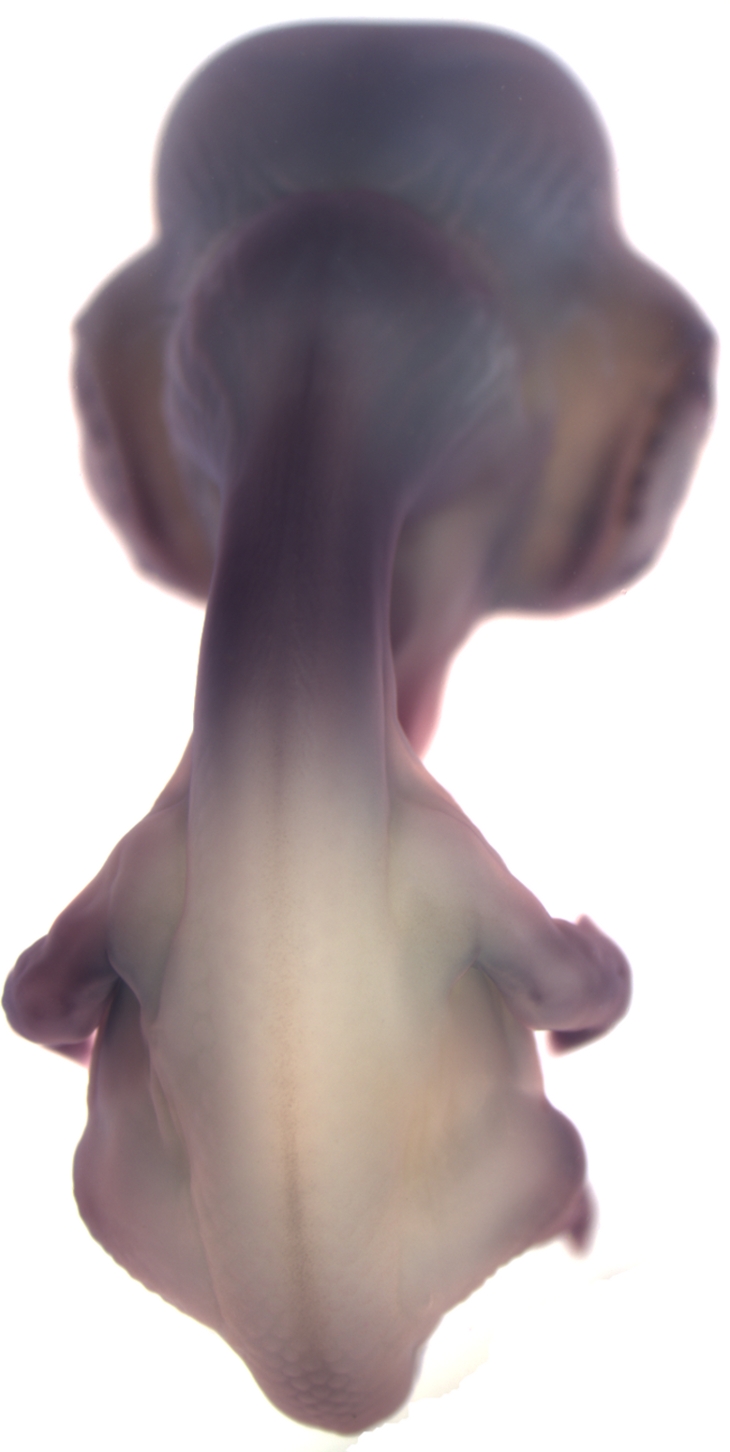
Expression of a retinoic acid–synthesizing enzyme (dark color) distinguishes neck from body skin in avian embryos, making the neck particularly sensitive to abolition of feather development.

Some birds such as vultures, ostriches, and certain species of stork have naked necks. This unusual feature allows them to tolerate heat better in hot climates. And, for vultures, the absence of neck feathers helps them poke about the insides of carrion unimpeded. The fossil record provides no evidence that naked necks evolved in a common ancestor, leaving scientists to explore other avenues to understand how bird neck feathering could have been lost independently in several bird species. A new study in *PLoS Biology* by Chunyan Mou, Denis Headon, and their colleagues provides clues to this mystery by investigating a mutation that affects skin patterning.

Patterns in vertebrate skin operate on a macro and micro level. In macropatterning, body parts may having strikingly different patterns of hair, scales, or feathers, such as a horse’s mane or a male peacock’s tail feathers. In micropatterning, the periodic spacing between individual hairs or feathers is typically uniform at any given location in the skin.

Unlike macropatterns, which are defined by positional information conveyed by the dermis distinct to different anatomical regions, micropatterns are controlled by so-called reaction-diffusion mechanisms in which opposing activating and inhibiting protein signals dictate whether or not a field of cells may give rise to skin appendages like hair or feathers. The stronger the activating signal and the weaker the inhibitory signal, the denser the appendages (and vice versa). Previous research in chickens and mice suggests that various bone morphogenetic proteins (BMPs) act as inhibitors in skin reaction-diffusion systems, while WNT and FGF pathway proteins have activating effects. Proteins in the BMP family are known to be involved in many developmental processes during embryogenesis, including early feather development.

Although the end result of reaction-diffusion systems in the skin is a field of cells that can give rise to hair or feathers of a certain density, by comparing the hairless tail of a mouse to its furry body or the bare neck of an ostrich to the feathers on its wings, it’s obvious that micropatterning isn’t uniform across the body. How differences in skin macropatterns translate to the micropattern level, leading to different densities of appendages, or even no appendages at all, remains obscure. To address this question, the authors analyzed a mutation in chickens aptly called Naked neck. In a previous study, the authors mapped the Naked neck mutation to a large region on chicken chromosome 3. This time, they used genetic fine mapping to narrow down the responsible region and found that of five candidate genes in the smaller region, one, *BMP12*, was normally expressed in embryonic skin and clusters of cells that will give rise to individual feathers (called placodes). Furthermore, *BMP12* expression was increased in the skin of Naked neck mutant embryos at the time when feather patterning begins. Further mapping revealed that a large DNA insertion 260,000 base pairs away from the *BMP12* gene was always present in chickens with the Naked neck mutation but never in wild-type chickens, indicating that it is associated with the Naked neck trait.

How might this mutation modulate feather patterning on neck skin? In chickens, the first feather macropattern occurs seven days after fertilization and consists of 14 stripes of cells that run along the length of the developing embryo. These cell stripes broaden and propagate on both sides of the body. Micropatterning follows close behind in the cell stripes, with the establishment of rows of placodes. After determining that BMP signaling is increased in Naked neck mutant embryos, the authors treated explant cultures of wild-type chicken skin with high levels of BMP12 protein and found that it caused loss of placodes on neck skin but not on body skin, recapitulating the Naked neck phenotype. This suggests that there is something about neck skin that makes it more sensitive to BMP signaling compared to skin on the body.

To figure out the molecular basis for neck skin’s higher sensitivity to BMP signaling, the authors compared the gene expression profiles of neck and body skin. This revealed that expression of a subset of genes involved in retinoic acid signaling was much higher in neck skin than in body skin. Although retinoic acid is known to play a role in determining the identity and orientation of feathers, it had not been implicated in micropatterning before. To see if this role was limited to chickens, the authors examined duck, turkey, quail, and guinea fowl embryos and found that they, too, had higher expression of retinoic acid target genes on neck compared to body skin.

Like BMP, treating skin explant cultures with retinoic acid inhibited placode formation, but unlike BMP, retinoic acid treatment inhibited feather development on both neck and body skin. This suggests that retinoic acid may act to sensitize the skin to the inhibitory effects of BMP signals, leading to complete loss of feathers on the neck where retinoic acid is highly produced.

The authors’ findings offer interesting insights into the developmental mechanisms responsible for the great variety of feather patterns seen in birds. The Naked neck mutation caused an increase in BMP expression throughout the skin of the body, but this led to major feathering changes only in the retinoic acid–defined developmental module comprising neck skin. These results demonstrate how mutations, which are expressed throughout the organism, can affect just part of an organism’s form. They also show how developmental modules can underlie significant steps in evolution, by obviating the need to generate a new trait like macropattern information from scratch.

**Mou C, Pitel F, Gourichon D, Vignoles F, Tzika A, et al. (2011) Cryptic Patterning of Avian Skin Confers a Developmental Facility for Loss of Neck Feathering. doi:10.1371/journal.pbio.1001028**

